# Riluzole But Not Melatonin Ameliorates Acute Motor Neuron Degeneration and Moderately Inhibits SOD1-Mediated Excitotoxicity Induced Disrupted Mitochondrial Ca^2+^ Signaling in Amyotrophic Lateral Sclerosis

**DOI:** 10.3389/fncel.2016.00295

**Published:** 2017-01-06

**Authors:** Manoj Kumar Jaiswal

**Affiliations:** Center of Physiology, University of GöttingenGöttingen, Germany

**Keywords:** ALS, SOD1^G93A^, riluzole, melatonin, mitochondria, excitotoxicity, cell death, Ca^2+^ signaling

## Abstract

Selective motoneurons (MNs) degeneration in the brain stem, hypoglossal motoneurons (HMNs), and the spinal cord resulting in patients paralysis and eventual death are prominent features of amyotrophic lateral sclerosis (ALS). Previous studies have suggested that mitochondrial respiratory impairment, low Ca^2+^ buffering and homeostasis and excitotoxicity are the pathological phenotypes found in mice, and cell culture models of familial ALS (fALS) linked with Cu/Zn-superoxide dismutase 1 (SOD1) mutation. In our study, we aimed to understand the impact of riluzole and melatonin on excitotoxicity, neuronal protection and Ca^2+^ signaling in individual HMNs *ex vivo* in symptomatic adult ALS mouse brain stem slice preparations and in WT and SOD1-G93A transfected SH-SY5Y neuroblastoma cell line using fluorescence microscopy, calcium imaging with high speed charged coupled device camera, together with immunohistochemistry, cell survival assay and histology. In our experiments, riluzole but not melatonin ameliorates MNs degeneration and moderately inhibit excitotoxicity and cell death in SH-SY5Y^WT^ or SH-SY5Y^G93A^ cell lines induced by complex IV blocker sodium azide. In brain stem slice preparations, riluzole significantly inhibit HMNs cell death induced by inhibiting the mitochondrial electron transport chain by Na-azide. In the HMNs of brainstem slice prepared from adult (14–15 weeks) WT, and corresponding symptomatic SOD1^G93A^ mice, we measured the effect of riluzole and melatonin on [Ca^2+^]_i_ using fura-2 AM ratiometric calcium imaging in individual MNs. Riluzole caused a significant decrease in [Ca^2+^]_i_ transients and reversibly inhibited [Ca^2+^]_i_ transients in Fura-2 AM loaded HMNs exposed to Na-azide in adult symptomatic SOD1^G93A^ mice. On the contrary, melatonin failed to show similar effects in the HMNs of WT and SOD1^G93A^ mice. Intrinsic nicotinamide adenine dinucleotide (NADH) fluorescence, an indicator of mitochondrial metabolism and health in MNs, showed enhanced intrinsic NADH fluorescence in HMNs in presence of riluzole when respiratory chain activity was inhibited by Na-azide. Riluzole’s inhibition of excitability and Ca^2+^ signaling may be due to its multiple effects on cellular function of mitochondria. Therefore formulating a drug therapy to stabilize mitochondria-related signaling pathways using riluzole might be a valuable approach for cell death protection in ALS. Taken together, the pharmacological profiles of the riluzole and melatonin strengthen the case that riluzole indeed can be used as a therapeutic agent in ALS whereas claims of the efficacy of melatonin alone need further investigation as it fail to show significant neuroprotection efficacy.

## Introduction

One of the hallmarks of ALS is extensive loss of MNs in the brain stem, hypoglossal nucleus, facial nucleus, and the spinal cord (Spalloni et al., 2004; Von Lewinski and Keller, 2005a,b; Spiller et al., 2016). Evidence is increasing that Ca^2+^ dysregulation and mitochondrial dysfunction is involved in the SOD1 G93A (SOD1^G93A^) mouse model (Kong and Xu, 1998; Jaiswal and Keller, 2009; Jaiswal, 2013, 2014; Chang and Martin, 2016; Paine and Jaiswal, 2016). The precise mechanisms leading to the selective loss of MNs in ALS patients as well as a possible determinant of selective MNs death in transgenic (Tg) mouse models of this disorder remain elusive. Many findings indicated two important characteristic features of this disorder: (1) the presence of low level of calcium binding proteins in combination with high buffering capacity in affected MNs (Alexianu et al., 1994; Kaal et al., 1998; Laslo et al., 2000; Jaiswal et al., 2009; Jaiswal, 2014) and (2) involvement of AMPA receptors (AMPA_R_; Rothstein, 1995; Van Den Bosch et al., 2000; Udagawa et al., 2015; Chang and Martin, 2016).

Riluzole is the only FDA-approved neuroprotective compound used in clinics for the treatment of ALS/MN disease (Bensimon et al., 1994; Jehle et al., 2000; Obinu et al., 2002; Gutierrez et al., 2016). Riluzole’s neuroprotective properties appear to arise from both presynaptic and postsynaptic mechanisms for attenuating glutamatergic neurotransmission, allowing riluzole to act as an antiglutamate drug (Bryson et al., 1996; Doble, 1996; Wokke, 1996; Geevasinga et al., 2016) and it block depolarization-evoked Ca^2+^ transients in MNs (Hubert et al., 1998). Moreover, many groups reported that riluzole works by inhibiting Na^+^ channels (Stefani et al., 1997; Yokoo et al., 1998; Zona et al., 1998; Geevasinga et al., 2016), inhibits Cl^-^ channels (Bausch and Roy, 1996), increases glutamate uptake and has an impact on glutamate receptors (Azbill et al., 2000) and inhibits GABA reuptake (Kretschmer et al., 1998). Additionally, riluzole was shown to attenuate neurotoxicity in tissue preparations and an animal model as well (Lang-Lazdunski et al., 2000; Mu et al., 2000). Earlier it was shown that an increase in [Ca^2+^]_i_ is a critical messenger for diverse pathophysiological events or in case of ALS and other neurodegenerative disorder and generally abnormal [Ca^2+^]_i_ increases are cytotoxic (Bootman et al., 1993; Berridge, 1997; Budd, 1998). Riluzole is known to block persistent sodium currents and earlier it was shown that riluzole block the Trpm4 (pore-forming subunits of Sur1-regulated NCCa-ATP channels), a key mechanism for the beneficial effect of riluzole in spinal cord injury (Simard et al., 2012). Moreover, Trpm4 is a Ca^2+^-activated cation channel that were found to expressed at higher levels in Th2 cells and inhibition of Trpm4 expression increased Ca^2+^ influx (Weber et al., 2010) via Ca^2+^ release-activated Ca^2+^ (CRAC) channel and Ca^2+^-activated K^+^ current (*K*_ca_) channels (Dolmetsch and Lewis, 1994). More recently it was discovered that Ca^2+^ influx and oscillations are also regulated by Trpm4 in Jurkat T cells (Launay et al., 2004). Work from all these previous studies indicated three compelling reasons for the beneficial effects of riluzole (i) block of persistent Na+ currents (molecular mechanism not known), (ii) inhibition of glutamatergic signaling pathways and (iii) an indirect neurotrophic effect. In spite of all these discoveries, it is not precisely established yet the precise mechanism through which riluzole acts on ion channels either directly or through second messenger signaling cascades, which attenuate ion influx-induced neuronal death.

Another effective cellular antioxidant known to be efficiently scavenging toxic free radicals, ROS and associated reactants is Melatonin (Jou et al., 2004; Reiter et al., 2016). Recent findings in which azides/CN-induced cell death via blocking complex IV of the mitochondrial ETC is reversed by melatonin and prevention of mitochondrial damage induced by ruthenium red (inhibits the mitochondrial Ca^2+^ uniporter for Ca^2+^ uptake), suggest that melatonin may act at the mitochondrial subcellular level and reduce ROS induced damage (Yamamoto and Tang, 1996; Martin et al., 2000). Furthermore, administrations of melatonin repair mitochondrial functioning in aging mice and shown to be neuroprotective in *in vitro* models of Alzheimer’s disease (Pappolla et al., 1997; Okatani et al., 2003).

Collectively, these results demonstrate the protective effects of riluzole and melatonin in neurodegenerative diseases involving mitochondrial dysfunction. However, we do not know if the therapeutic effects of these drugs in various pre-clinical mitochondria related neurodegenerative diseases also effective in ALS. Moreover, since mitochondrial ROS-induced cell death and excitotoxicity play a prominent role in ALS disease, specific neuroprotection mechanisms of riluzole and melatonin at the mitochondrial sub cellular level involving excitotxicity and Ca^2+^ signaling need to be further investigated. In this context, we determine whether treatment with riluzole or melatonin could attenuate sodium-azide (Na-azide) induced ROS and eventually cell death. Additionally, we investigated the impact of riluzole and melatonin on vulnerable HMNs of adult symptomatic SOD1^G93A^ mice and corresponding wild type (WT) littermates. Imaging experiments were performed on adult brain stem slices where mitochondrial function of HMNs was disturbed by a bath application of 3 mM Na-azide, which inhibits complex IV and thereby disturbs mitochondrial metabolism. Riluzole and melatonin protection studies were similarly designed where application of sodium azide was performed because of, (1) its quick and reversible action, and (2) In both sALS and fALS a decrease in mitochondrial complex IV observed (Nowicky and Duchen, 1998; Menzies et al., 2002). The cell death, cell survival and Ca^2+^ signals were characterized and evaluated in the presence or absence of drugs.

We found that riluzole moderately ameliorates MN degeneration, inhibits excitotoxicity and mildly and reversibly inhibits Ca^2+^ signaling. However, melatonin fails to show significant MN protection and significant impact on Ca^2+^ signaling both in adult WT and corresponding SOD1^G93A^ mice as well as in cell culture model of ALS. We anticipate that this experimental system can be used to screen the drugs targets either alone or in combination of drug cocktails that can be used for large-scale compound screening.

## Materials and Methods

### SH-SY5Y^WT^ and SH-SY5Y^G93A^ Neuroblastoma Cell Lines

SH-SY5Y human neuroblastoma cell line transfected with either WT or G93A mutant linked with fALS were routinely cultured in growth medium and were used as a valid and robust *in vitro* system to investigate the cellular excitotoxicity and mitochondria mediated alterations associated with SOD1-G93A mutations (Carri et al., 1997; Goos et al., 2007; Jaiswal et al., 2009). Detail descriptions about the transfection strategy, cell culture maintenance, cell culture growth medium and procedures are described earlier (Jaiswal et al., 2009).

### Measurement of Cell Viability Using Mitochondrial Toxin Sodium Azide and Neuroprotection by Riluzole and Melatonin

Toxicity of Na-azide induced mitochondrial inhibition and thereby cell death assay in SH-SY5Y^WT^ and SH-SY5Y^G93A^ was done using bright field microscopy, hematoxylin and eosin (H&E) histochemistry and WST-1 assay. Neuroprotection study was done using similar method. SH-SY5Y^WT^ and SH-SY5Y^G93A^ transfected cells on glass coverslips were treated for 3 min (acute), 30 min (moderate) and 60 min (chronic) exposure with 3 mM Na-azide (60 min data not shown), cells were fixed with 4% paraformaldehyde (PFA), dehydrated through water/ethanol and histolene and then stained with Meyer’s hemalum reagent (1:1 in H_2_O). While choosing the Na-azide concentrations we followed the previous literature and accordingly we selected three different Na-azide concentrations, 1, 3, and 10 mM for cytotoxicity induced cell death assays and neuroprotection study (Hunter et al., 1959; Harvey et al., 1999). Concentration of 1 mM Na-azide not always leads to swelling of mitochondria for acute (3 min) exposure. Also shown by other groups at 0.1 mM or less than 2 mM sodium cyanide (NaCN) was not always adequate to prevent completely spontaneous swelling or swelling induced by low concentrations of phosphate (Hunter et al., 1959; Harvey et al., 1999). Higher concentration, e.g., 10 mM Na-azide might react with components other than cytochrome oxidase, therefore based on our pilot toxicity experiments and previous literature, we have chosen 3 mM Na-azide concentration suitable for our acute toxicity (3 min exposure) and neuroprotection assays. To study the differences in impact of Na-azide–induced cell death and neuroprotection mediated by riluzole and melatonin, SY5Y^WT^ and SH-SY5Y^G93A^ cells at a density of 10^5^ cells/cm^2^ were seeded into 24-well plates. SH-SY5Y^WT^ and SH-SY5Y^G93A^ cells were exposed to culture medium with 3 mM Na-azide for 3 min (acute), 30 and 60 min (chronic). After 3, 30, and 60 min (60 min data not shown) of exposure, cell photomicrograph was acquired using brightfield microscope. Furthermore, for neuroprotection assay by riluzole and melatonin, both cell lines were exposed to DMEM growth medium with 3 mM Na-azide+100 μM riluzole or 3 mM Na-azide+100 μM melatonin for 3 min (acute). After rinsing the cover slips twice with water, cover slips were dehydrated and Vectashield Medium (Vector Laboratories, Burlingame, CA, USA) was used for mounting the cover slips.

To further investigate the impact of Na-azide exposure on cell viability and neuroprotection by riluzole and melatonin, cells at a density of 10^5^ cells/cm^2^ in 24-well plates were treated with culture medium or medium containing 3 mM Na-azide final concentration. After 3, 30, and 60 min (60 min data not shown) of incubation with 3 mM Na-azide, WST-1 cell proliferation reagent (Roche Applied Science, Mannheim, Germany) was used to determine cell viability. For neuroprotection, similar WST-1 assay were done using 3 mM Na-azide+100 μM riluzole or 3 mM Na-azide+100 μM melatonin. The WST-1 assay is comprises a cleavage of the tetrazolium salt by healthy mitochondria to soluble orange formazan, by the enzyme succinate dehydrogenase present in the ETS of healthy mitochondria. The formazan dye formed after incubation of cells with WST-1 for 2 h was quantified by measuring the absorbance at 490 nm using the Genios multiplate reader (Tecan, Crailsheim, Germany) and correlates with the total number of metabolically viable cells.

### Animals and Genotyping

Superoxide dismutase 1^G93A^ Tg 1Gur (Fast Line) mice strain is considered a well-established animal model for human ALS. This mice strain acquired from the Jackson Laboratory (Bar Harbor, ME, USA) and in-house breeding was carried out in our animal facility. The SOD1^G93A^ Tg mouse develop paralysis in the limb and eventually dies at 4–5 months of age due to the loss of MNs in the brain stem, HMNs, FMNs, and the spinal cord whereas WT littermates were unaffected. The Tg mouse carries a variation of the human mutant G93A-SOD1 gene in which at position 93 glycine is substituted by alanine (Gurney et al., 1994). Breeding and genotyping procedures were adapted from Hoyaux et al. (2000; for further details also see Jaiswal and Keller, 2009). Animal breeding and experimental procedures were approved and carried out in accordance with the guidelines of the ethics committee of the Medical Faculty of Georg-August University, Göttingen, Germany.

### Preparation of Acute Brain Stem Slices from Adult SOD1^G93A^/WT Mice Littermates

Mice were anesthetized until the paw withdrawal reflex disappeared in a chamber containing an ether vapor-enriched atmosphere and immediately sacrificed. Mice brains were quickly removed and then put in 4°C ice-cold aCSF. Transverse 250 μm thick acute brain stem slices were prepared from the 14–15 weeks old WT and symptomatic SOD1^G93A^ mice in late stage of motor dysfunction according to the procedures described earlier (Ladewig and Keller, 2000; Jaiswal and Keller, 2009) using a vibroslicer (Leika VT 1000S, Göttingen, Germany). Selectively vulnerable brain stem HMNs (Nuc. hypoglossus; XII) region were visually identified by their location nearby to the IV ventricle during the brain stem slice preparations transversely (**Figure 3**). To achieve maximum oxygen supply to slices, aCSF (in mM: NaCl 118, KCl 3, NaH_2_PO_4_ 1, CaCl_2_ 1.5, MgCl_2_ 1, NaHCO_3_ 25, glucose 20; pH 7.4; 320 mOsm) was constantly simmered with a mixture of 95% O_2_ and 5% CO_2_ (carbogen). An essential requirement for the microfluorometric measurements was the preservation of MNs in adult slices near to the slice surface in a physiologically viable and intact condition. This challenging requirement for Ca^2+^ imaging in MNs was accomplished by reducing mechanical tissue disturbances during the thick adult brain slice preparations, slice cutting at 4°C temperature and regular maximum oxygen delivery to keep metabolic conditions optimum for cells viability (For details, see Jaiswal and Keller, 2009). Slices were kept at ∼27°C in continuous carbogen-bubbled aCSF solution at pH 7⋅4 for 30 min and then let it cool down to room temperature (RT, ∼20–21°C) prior to dye loading. Unless indicated otherwise, all experiments were performed at RT.

### Charge Coupled Device (CCD) Fast Optical Imaging

Intracellular calcium [Ca^2+^]i fluorescence measurement were achieved using an optical recording system including an upright Axioscope microscope (Zeiss, Göttingen, Germany) equipped with a computer-operated monochromator (TILL Photonics, Martinsried, Germany) built on a galvanometric scanner (Polychrome II, TILL Photonics, Martinsried, Germany). Briefly, a modified version of the CCD camera system (TILL Photonics, Planegg, Germany) and Achroplan W 63×, 0.9W objective were used to collect fluorescent signal changes in defined “regions of interest” (ROIs) in cell soma. Fluorescence signals changes were digitized using a 12-bit CCD camera (PCO, Kelheim, Germany), binning of which was put to 4 × 4, with a sampling rate between 3 and 13 Hz using TILL Vision Software (TILL Vision Software V3.3, TILL Photonics, Germany). Dichroic mirror with mid reflection at 425 nm was used for Fura-2 AM calcium dye (Invitrogen, Carlsbad, CA, USA) to direct the emitted light. To achieve the ratiometric calcium imaging, swapping between wavelengths (360 and 390 nm) was achieved in ∼3 ms, thereby allowing fast ratiometric Ca^2+^ measurements.

### Microfluorometric Ca^2+^ Measurements in SH-SY5Y Transfected Cells and HMNs from WT and SOD1^G93A^ Brain Stem Slice Preparations

WT (SH-SY5Y^WT^) and SOD1 (SH-SY5Y^G93A^) transfected cell layers on cover slips were stained with membrane-permeable Fura-2 AM (*K*_d_ ∼ 0.2 μM) dye by incubating with RPMI-1640 medium having 10% FCS (Invitrogen, Carlsbad, CA, USA) and 0.846 mM Ca^2+^ (5 μM Fura-2 AM dissolved in DMSO containing 10% pluronic acid for 30 min at 37°C and continuous bubbled with 95% O_2_ and 5% CO_2_). After the dye incubation cells were washed with RPMI and incubated in culture medium for ∼20 min at 37°C to permit de-esterification of dye. Baseline [Ca^2+]^i were measured in transfected cells 2–5 days in culture. Chemical induction of Ca^2+^ measurements were done by incubating the cells for 3 min with 3 mM Na-azide and effects of 100 μM riluzole and 100 μM melatonin were measured by inhibition of Ca^2+^ with similar measurements (data not shown).

In HMN_S_ of brain stem slice, calcium signals were measured by defining appropriate ROIs of MN somas as previously described (Lips and Keller, 1998). [Ca^2+^]_i_ signals were monitored by bath-loading of the brain stem slices with 5 μM final dye concentration of Fura-2 AM for 40 min at 27°C with continuous carbogen bubble (5% CO_2_ and 95% O_2_). After the dye incubation, prior to onset of imaging slices were washed with aCSF and incubated another 30 min in aCSF for de-esterification of Fura-2 AM. Excitation of Fura-2 was done alternately at 360 and 390 nm by UV light and emitted light was directed to a dichroic mirror having mid-reflection at 425 nm and filtered by a band pass filter of 505–530 nm (Zeiss, Goettingen, Germany) using a computer-controlled monochromator. Changes in [Ca^2+^]_i_ are denoted as variations in Fura-2 ratio (*F*/*F*_0_) where *F* = fluorescence at different time points of experiment and *F*_0_ = baseline fluorescence value before drug/chemical applications (for details see Jaiswal and Keller, 2009). Further calculations and analysis of [Ca^2+^]_i_ signals were performed oﬄine using IGOR Pro (Wavemetrics, Lake Oswego, OR, USA) and OriginPro 6.0 (OriginLab Corporation, Northampton, MA, USA) Software.

### Intrinsic nicotinamide adenine dinucleotide (NADH) Fluorescence

The metabolic state of HMNs from SOD1^G93A^ mice in the presence and absence of 100 μm riluzole was monitored by measuring and monitoring intrinsic nicotinamide adenine dinucleotide (NADH) fluorescence of HMNs in the slice preparations using a high-speed CCD camera. Briefly, NAD(P)H excitation at 360 nm was carried via a computer-controlled monochromator (TILL Photonics, Martinsried, Germany) reflected onto the surface of the slice using dichroic mirror (400 nm, Zeiss, Goettingen, Germany). The hypoglossal nucleus was easily recognized by a high level of intrinsic fluorescence. Fluorescence emission was collected by using a CCD camera (TILL Photonics, Planegg, Germany). All experiments used a 410 nm LP filter between the dichroic mirror and camera to maximize light capture. Imaging was performed after focusing onto the surface of slices in hypoglossal area using 63× (0.9 NA) Achroplan water objective (Zeiss, Göttingen, Germany) and collected by a 12-bit CCD camera (PCO, Kelheim, Germany) described earlier. Calculations and analysis of NAD(P)H signals were performed oﬄine after the experiment using IGOR Pro (Wavemetrics, Lake Oswego, OR, USA) and OriginPro 6.0 (OriginLab Corporation, Northampton, MA, USA) Software.

### Chemical Reagents

Fura-2 AM dye (50mg/vial) was purchased from Invitrogen (Carlsbad, CA, USA) and dissolved in 50 μL DMSO containing 20% Pluronic F-127 to a concentration of 1 mM. Na-azide, riluzole, melatonin and all other chemicals were purchased from Sigma–Aldrich (St. Louis, MO, USA). To prepare the stock solutions, riluzole and melatonin were dissolved in 100% ethanol to make 10 mM stock solutions and all the time kept at 4°C to avoid ethanol evaporation. Na-azide was dissolved in distilled water. To keep cells viable drug solutions when mixed in the perfusate always simmered with carbogen (95% O_2_, 5% CO_2_) during the experiments.

### Statistical Analysis

SH-SY5Y^WT^ and SH-SY5Y^G93A^ transfected cells attached with cover slip was used for single experiments and replicate in five separate experiments (five field of view) for each condition in cell viability assay (cell survival/death) and seven separate experiments (seven field of view for each cover slides for SH-SY5Y^WT^ and SH-SY5Y^G93A^) for neuroprotection assay. All slices were used for a sole experiment and 5–6 HMNs taken from each slice in Fura-2 calcium imaging data experiments. 2–3 slices were used per mouse and 5–6 HMNs selected from each slice for bar graph presentation. In slice experiments, bar diagram represents 12 imaging experiments recorded from 12 separate slices obtained from 5 WT mice and 10 imaging experiments recorded from 10 slices obtained from 5 SOD1^G93A^ mice in acute Na-azide experiments. In case of riluzole experiments, bar diagram represent 14 imaging experiments recorded from 14 separate slices obtained from 6 WT mice and 13 imaging experiments recorded from 13 slices obtained from 6 SOD1^G93A^ mice. In case of NADH fluorescence imaging, 5–6 cells from slice for five separate experiments for each condition from five different mice of same genotypes was used for bar diagram representation. Values in the text and error bars in experimental figures are represented as mean ± SD. Significance for all the *in vitro* assay, slice imaging and pharmacological intervention was calculated using the two-tailed unpaired student *t*-test and a *p*-value < 0.05 was considered statistically significant. One-way analysis of variance (ANOVA) with Bonferroni’s method was used as the *post hoc* test used to recognize statistically significant Na-azide effects, and neuroprotection effects of riluzole and melatonin exposed cells as compared with controls with in groups.

## Results

### Cell Death Assessment by Inhibition of Mitochondria and Neuroprotective Actions of Riluzole and Melatonin against Sodium Azide-Induced Cell Death in *In vitro* SH-SY5Y Cell Culture Model of ALS

**FIGURE 1:**
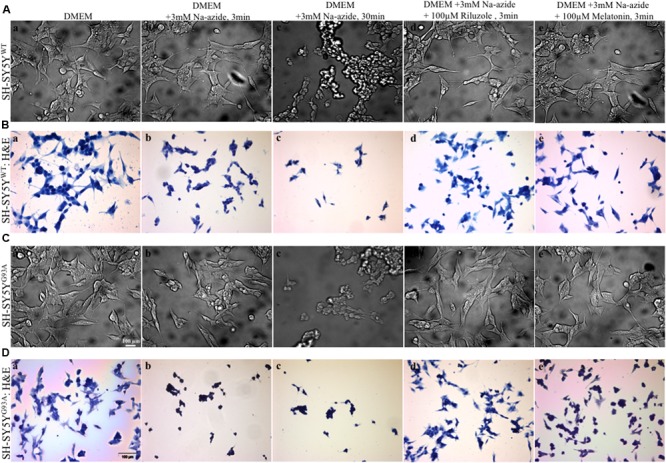
**Motor neurons cell death assessment using mitochondrial toxin Sodium azide and neuroprotection assay to access efficacy of riluzole and melatonin. (A)** Photomicrograph of bright field images of differentiated SH-SY5Y^WT^ cells exposed to growth medium DMEM **(a)**, DMEM+3 mM Na-azide for 3 min **(b)**, DMEM+3 mM Na-azide for 30 min **(c)**, DMEM+3 mM Na-azide+3 mM riluzole for 3 min **(d)**, and DMEM+3 mM Na-azide+3 mM melatonin for 3 min **(e)**. Note the decrease in cell density after Na-azide exposure, which is ameliorated in presence of riluzole, but not melatonin. **(B)** H&E staining showed a substantially higher density of living SH-SY5Y^WT^ cells exposed to DMEM **(a)** compared to SH-SY5Y^WT^ cells exposed to DMEM+3 mM Na-azide for 3 min (acute, **b**) and 30 min (chronic, **c**). Note the shrinkage, clustering, and reductions in cell number of severely damaged/dead SH-SY5Y^WT^ neuroblastoma cells in **b** and **c**. H&E staining showed 3 min acute exposure of 3 mM Na-azide induced cell death is partially ameliorated in the presence of 3 mM riluzole **(d)**, but not in the presence of 3 mM melatonin **(e)**. **(C)** Bright field images of differentiated SH-SY5Y^G93A^ cells exposed to DMEM **(a)**, DMEM+3 mM Na-azide for 3 min **(b)**, DMEM+3 mM Na-azide for 30 min **(c)**, DMEM+3 mM Na-azide+3 mM riluzole for 3 min **(d)**, and DMEM+3 mM Na-azide+3 mM melatonin for 3 min **(e)**. Note the decrease in cell density in presence of 3 mM (acute) and 30 min (chronic) Na-azide exposure. Note the increase in cell density of SH-SY5Y^WT^ cells compared to SH-SY5Y^G93A^ cells in presence of 3 mM riluzole suggesting greater susceptibility of SH-SY5Y^G93A^ to mitochondrial-induced damage. **(D)** H&E staining showed a similar substantially higher density of living SH-SY5Y^G93A^ cells like SH-SY5Y^WT^ cells exposed to DMEM **(a)** compared to cells exposed to 3 min (acute, **b**) and 30 min (chronic, **c**) exposure with 3 mM Na-azide. Cell death of H&E stained SH-SY5Y^G93A^ cells in presence of 3 mM Na-azide exposure is partially ameliorated in presence of 3 mM riluzole **(d)**, but not in presence of 3 mM melatonin exposure **(e)**. Scale bar for **(A)** and **(C)** = 20 μm and **(B)** and **(D)** = 100 μm.

**FIGURE 2:**
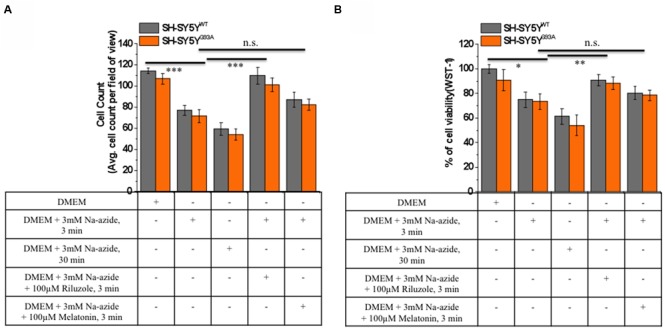
**Riluzole but not melatonin stimulation increased cells survival and neuroprotective against sodium azide induced mitochondrial ETC blockade both in SH-SY5Y^WT^ and SH-SY5Y^G93A^ cells. (A)** Live cell count of SH-SY5Y^WT^ (gray bar) and SH-SY5Y^G93A^ (red bar) neuroblastoma cells after incubation with DMEM, DMEM+3 mM Na-azide for 3 min, DMEM+3 mM Na-azide for 30 min, DMEM+3 mM Na-azide+3 mM riluzole for 3 min and DMEM+3 mM Na-azide+3 mM melatonin for 3 min (average of cell counts for seven field of view in each conditions). Please note the significant difference between riluzole-treated and non-treated SH-SY5Y^WT^ and SH-SY5Y^G93A^ cells exposed to Na-azide (*p* < 0.005). **(B)** Toxicity of Na-azide for SH-SY5Y^WT^ and SH-SY5Y^G93A^ cells was measured using the WST-1 cell viability test in presence of DMEM, DMEM+3 mM Na-azide for 3 min, DMEM+3 mM Na-azide for 30 min, DMEM+3 mM Na-azide+3 mM riluzole for 3 min and DMEM+3 mM Na-azide+3 mM melatonin for 3 min. SH-SY5Y^WT^ and SH-SY5Y^G93A^ cells were more vulnerable to the action of Na-azide in absence of riluzole compared to 3 mM riluzole presence in DMEM growth medium (*p* < 0.01). Data are expressed as the mean ± SEM. ^∗∗∗^*P* < 0.005, ^∗∗^*P* < 0.01, ^∗^*P* < 0.05 two-tailed unpaired student *t-*test.

We utilized differentiated SH-SY5Y^WT^ and SH-SY5Y^G93A^ cells used previously to generate WT and SOD1^G93A^ cell culture model of ALS (Jaiswal et al., 2009). Cell death, cell viability and anti-aggregate formation were assessed using bright field microscopy, immunocytochemistry and WST-1 cell proliferation assay (**Figures 1** and **2**). SH-SY5Y^G93A^ cells showed slightly higher levels of cell death compared to SH-SY5Y^WT^ after 3 and 30 min (**Figures 1A–Da–c** and **2A**) exposure to mitochondrial toxin Na-azide (**Figures 1A–Da–c** and **2A**). A similar pattern was observed while investigating the apoptosis and aggregation assessed by bright field microscopy demonstrating typical apoptotic morphology, e.g., condensation/fragmentation of nuclear material and elongation of cell size and cell membranes. Cells displaying fragmentation of nuclear materials (pyknosis)/cell membrane elongation were classified as apoptotic and showed aggregates formation with both cell lines after incubation for 30 and 60 min (**Figures 1Aa–c,Ca–c**; data not shown for 60 min exposure) but not with acute 3 min (acute) induction (**Figures 1Aa–c,Ca–c**). We have not quantified the cell numbers after 30 and 60 min exposure to Na-azide due to lump formation after 30 min exposure and inability to separate each cells with in aggregates and almost complete cell death 60 min after the inhibition of mitochondria by Na-azide (**Figures 1Ac,Cc**). Cell survival/death was assessed by H&E staining and WST-1 test. Cell count for H&E staining for both SH-SY5Y^WT^ and SH-SY5Y^G93A^ cells treated with Na-azide for 3 min (77.0 ± 4.7 for WT and 71.5 ± 6.1 for SOD1^G93A^) and 30 min (59.2 ± 5.8 for WT and 54.0 ± 5.5 for SOD1^G93A^) were significantly lower compared to untreated cells with Na-azide (114.5 ± 2.8 for WT and 107.0 ± 5.1 for SOD1^G93A^; **Figures 1A–Da–c** and **2A**; *N* = 5; ^∗∗∗^*P* < 0.001, ^∗∗^*P* < 0.005, Students two sample *t*-test, **Table 1**).

**Table 1:** Cell count of H&E staining for SH-SY5Y^WT^ and SH-SY5Y^G93A^ cells treated with sodium azide in presence and absence of riluzole and melatonin.

Treatment	SH-SY5Y^WT^	SH-SY5Y^G93A^	*P*-value
DMEM, 3 min	114.5 ± 2.84	107 ± 5.01	0.001
DMEM+3 mMNa-azide, 3 min	77 ± 4.79	71.5 ± 6.19	0.005
DMEM+3 mM Na-azide, 30 min	59.25 ± 5.83	54 ± 5.59	0.001
DMEM+3 mM Na-azide+100 μM Riluzole, 3 min	110 ± 7.85	101.25 ± 6.71	0.005
DMEM+3 mM Na-azide+100 μM Melatonin, 3 min	87.25 ± 7.09	82.5 ± 5.3619	n.s.

It was previously reported by several groups that riluzole and melatonin ameliorates ALS progression in SOD1^G93A^ mouse model and extend morbidity of hALS patients. We next attempted to determine whether riluzole and melatonin confers neuroprotection and ameliorate cell death in ALS cell culture model and if yes at what extent. Previously it was shown that riluzole up to 10 μM have no effect on cell survival in DA neurons and SH-SY5Y cells. However, concentrations greater than 50 μM have a remarkable effect on cell survival (Storch et al., 2000); therefore, here we used 100 μM riluzole and melatonin exposure with superfusate and evaluate the protective effects of riluzole on cell survival in SH-SY5Y^WT^ and SH-SY5Y^G93A^ cells *in vitro* (**Figures 1A–Dd,e** and **2A**). Our findings show that the acute inhibition of mitochondrial metabolism and function and thereby cell death by Na-azide is ameliorated by riluzole but there is no significant effects of melatonin in both SH-SY5Y^WT^ and SH-SY5Y^G93A^ cells (**Figures 1A–Dd,e** and **2A**). The average number of H&E positive cells in a given field of view (seven field of view) after the treatment of 100 μM riluzole to SH-SY5Y^WT^ and SH-SY5Y^G93A^ cells is significantly higher (**Figure 2A**; 110.0 ± 7.8 for WT and 101.2 ± 6.7 for SOD1^G93A^) compared to non-treated cells stimulated with 3 mM Na-azide (**Figure 2A**; 77.0 ± 4.7 for WT and 71.5 ± 6.1 for SOD1^G93A^; for riluzole treated and non-treated cells; ^∗∗^*P* < 0.005, Students two sample *t*-test; **Table 1**). In addition to decreasing the cell death, riluzole had significant protective effects on the neuritis, fine cell processes and cell membrane integrity being preserve from mitochondrial inhibition and the effect was most pronounced in SH-SY5Y^WT^ compared to SH-SY5Y^G93A^ cells (**Figures 1Ad–Dd**). However, impact of 100 μM melatonin treatment on Na-azide induced inhibition of mitochondria is not significantly different then non-treated cells in both SH-SY5Y^WT^ and SH-SY5Y^G93A^ cells though there are minor increase in cell count of WT and SOD1^G93A^ cells (**Figures 1A–De** and **2A**; 87.2 ± 7.0 for WT and 82.5 ± 5.3 for SOD1^G93A^, **Table 1**).

Cell viability assessed by WST-1 assay showed significant dose dependent ROS-induced mitochondrial toxicity and cell death. Cell viability of SH-SY5Y^WT^ and SH-SY5Y^G93A^ cells in presence of Na-azide for 3 and 30 min leads to a decrease in cell survival (75.0 ± 3.4 and 61.2 ± 6.1% for 3 and 30 min incubation, respectively, for WT; **Figure 2B**) and were significantly not different with SOD1^G93A^ cells (73.5 ± 6.2 and 54.0 ± 8.4% for 3 and 30 min incubation, respectively; **Figure 2B**; **Table 2**). However, cell viability for both WT and SOD1^G93A^ treated cells were much lower and significantly different compared to untreated cells without Na-azide toxicity (100 ± 3.4% for WT and 91 ± 8.6% for SOD1^G93A^; **Figures 1A–Da–c** and **2A**; *N* = 5; ^∗∗∗^*P* < 0.001, Students two sample *t*-test; **Table 2**). Similarly % of SH-SY5Y^WT^ and SH-SY5Y^G93A^ viable cells after the treatment of 100 μM riluzole is significantly higher (**Figure 2B**; 91.0 ± 4.5 for WT and 88.2 ± 5.2 for SOD1^G93A^; **Table 2**) compared to non-treated cells stimulated with 3mM Na-azide for 3 min (**Figure 2A**; 75 ± 6.3% for WT and 73.5 ± 6.3% for SOD1^G93A^; ^∗∗^*P* < 0.01, Students two sample *t*-test; **Table 2**). There is no significant increase in cell survival after melatonin treatment (**Figure 2B**; 80.2 ± 5.3 for WT and 78.5 ± 4.2 for SOD1^G93A^; **Table 2**).

**Table 2:** WST-1 cell viability assay in SH-SY5Y^WT^ and SH-SY5Y^G93A^ cells treated with sodium azide in presence and absence of riluzole and melatonin.

Treatment	SH-SY5Y^WT^	SH-SY5Y^G93A^	*P*-value
DMEM, 3 min	100 ± 3.42	91 ± 8.67	0.05
DMEM+3 mM Na-azide, 3 min	75 ± 6.34	73.5 ± 6.28	0.01
DMEM+3 mM Na-azide, 30 min	61.25 ± 6.17	54 ± 8.37	0.001
DMEM+3 mM Na-azide+100 μM Riluzole, 3 min	91 ± 4.51	88.25 ± 5.20	0.01
DMEM+3 mM Na-azide+100 μM Melatonin, 3 min	80.25 ± 5.34	78.5 ± 4.19	n.s.

### Impact of Riluzole on Sodium Azide-Induced Mitochondrial [Ca^2+^]_i_ Release and Excitotoxicity

**FIGURE 3:**
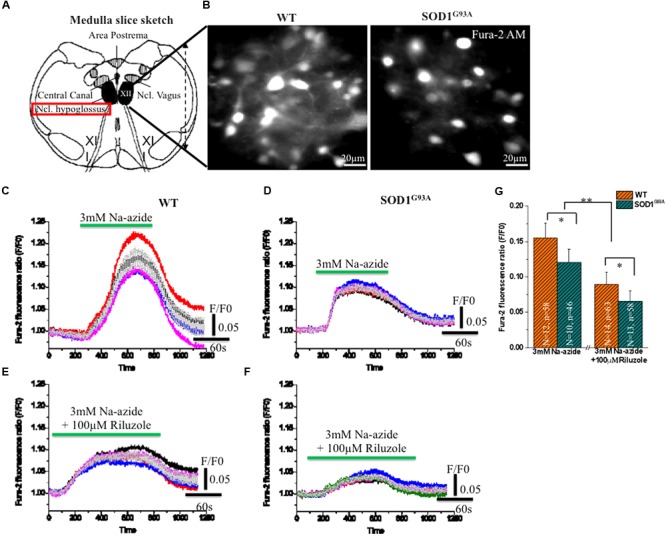
**Riluzole induce inhibition of [Ca^2+^]_i_ in fura-2 AM loaded HMNs exposed to 2 mM Na-azide in adult WT and symptomatic SOD1^G93A^ mice. (A)** A graphical illustration of the adult mouse brainstem slice containing hypoglossal motoneurons (HMNs) marked with black colors [Modified from Jaiswal, 2009. Optical analysis of [Ca^2+^]i and mitochondrial signaling pathways: implications for the selective vulnerability of motoneurons in amyotrophic lateral sclerosis (ALS). Goöttingen, Univ., Diss., 2008. Copyright© 2009 Jaiswal, M. K. ediss.uni-goettingen.de. Used with permission]. **(B)** A CCD camera images (4 × 4 binning) showing 15 weeks adult WT (left) and symptomatic SOD1 (right) mice HMNs stained with ratiometric calcium dye fura-2 AM (excitation at 360 nm). **(C)** 3 mM Na-azide was added in aCSF superfusate and the calcium fluorescence signal was recorded in 14–15 weeks old WT mice HMNs. **(D)** 3 mM Na-azide was added in aCSF superfusate and the calcium fluorescence signal was recorded in 14–15 weeks old symptomatic SOD1^G93A^ mice HMNs. **(E)** 3 mM Na-azide was added in aCSF superfusate together with 100 μM riluzole and the calcium fluorescence signal was recorded in 14–15 weeks old WT mice HMNs. **(F)** 3 mM Na-azide was added in aCSF superfusate together with 100 μM riluzole and the calcium fluorescence signal was recorded in 14–15 weeks old symptomatic SOD1^G93A^ mice HMNs. Average peak amplitude (gray color) shown as means from 5 to 6 cells in an imaging field **(C–F)**. **(G)** Bar diagram of effect of 100 μM riluzole on Na-azide induced blockade of [Ca^2+^]_i_ measured in adult WT (Light gray) and symptomatic SOD1^G93A^ (Gray sparse) mice HMNs. Data are expressed as means 12 imaging experiments from five WT mice and corresponding 10 imaging experiments from five symptomatic SOD1^G93A^ mice in acute 3 mM Na-azide experiments. In case of riluzole experiments, bar diagram represent 14 imaging experiments obtained from six WT mice and corresponding 13 imaging experiments obtained from six symptomatic SOD1^G93A^ mice. The changes in peak amplitude of [Ca^2+^]_I_ were calculated as *F*/*F*_0_ (360/390). ^∗^*P* < 0.05, ^∗∗^*P* < 0.01 compared with adult WT and symptomatic SOD1^G93A^ mice after riluzole application. Data are expressed as the mean ± SD.

To test further the efficacy of riluzole on Na^+^-azide induced mitochondrial Ca^2+^ signaling, Fura-2 AM stained HMNs of brain stem slices were exposed to Na-azide in presence and absence of 100 μM riluzole. Sketch diagram of hypoglossal MNs in brainstem slice preparations (**Figure 3A**) and representative photomicrograph of fura-2 AM stained HMNs in WT and (left) and symptomatic SOD1^G93A^ mice (right) shown in **Figure 3B**. We evaluated the protective impact of riluzole on calcium signaling of HMNs of adult WT (**Figures 3C,E**) and symptomatic SOD1^G93A^ mice brain stem slices *in vitro* (**Figures 3D,F**). We found that riluzole has a moderate effect on inhibition of Na-azide (3 mM) induced [Ca^2+^]_i_ signaling of MN’s mediated by Na^+^-azide induced excitotoxicity. At higher concentration (>50 μM) riluzole moderately inhibited [Ca^2+^]_i_ fluorescence in both WT and selectively impaired HMNs of symptomatic SOD1^G93A^ mice whereas at lower concentrations (<50 μM) there is no significant effect (data not shown). Sodium azide-induced mitochondrial [Ca^2+^]_i_ release response is significantly different between WT and symptomatic SOD1^G93A^ mice. The impact of riluzole inhibition is not specific to only SOD1^G93A^ mice and it also moderately inhibits [Ca^2+^]_i_ signaling in WT mice as well (**Figure 3**; **Table 3**).

**Table 3:** Sodium azide-induced mitochondrial [Ca^2+^]_i_ release and excitotoxicity in absence and presence of riluzole.

Fura-2 fluorescence intensity (*F*/*F*_0_)	WT	SOD1^G93A^	*P*-value
3 mM Na-azide	0.15 ± 0.020	0.11 ± 0.018	0.05
3 mM Na-azide+100 μM Riluzole	0.089 ± 0.017	0.065 ± 0.014	0.01

As illustrated in **Figures 3C,D,G**; 3 mM Na-azide caused a rise in the [Ca^2+^]_i_ transient by mitochondrial complex IV inhibition, with average peak amplitudes (*F*/*F*_0_) reaching 0.15 ± 0.02 and 0.11 ± 0.01 (**Figures 3C,D,G**; **Table 3**) respectively, in HMNs of adult WT (5 mice/*N* = 12 slice; *n* = 58 HMNs) and symptomatic SOD1^G93A^ mice (5 mice/*N* = 10 slice; *n* = 46 HMNs) and subsequently comes to baseline over 2–3 min wash out with aCSF. In the presence of 100 μM riluzole, application of 3 mM Na-azide caused a rise in the [Ca^2+^]_i_ transient, with average of the peak amplitudes (*F*/*F*_0_) reaching 0.089 ± 0.01 and 0.065 ± 0.01 (**Figures 3E–G**; **Table 3**) respectively, in HMNs of adult WT (6 mice/*N* = 14 slice; *n* = 63 HMNs) and symptomatic SOD1^G93A^ mice (6 mice/*N* = 13 slice; *n* = 58 HMNs). The inhibition of the peak amplitude of [Ca^2+^]_i_ in SOD1^G93A^ mice (*F*/*F*_0_ = 0.065 ± 0.01) is prominent compared to WT mice (*F*/*F*_0_ = 0.089 ± 0.01; *P* < 0.05, Students two sample *t*-test; quantitative fluorescence value compared in **Table 3**). The inhibition of [Ca^2+^]_i_ peak amplitude in SOD1^G93A^ mice by riluzole might be attributed to a decrease in the entry of Ca^2+^ through a voltage dependent calcium channel (VDCC) and need further investigations. We conclude that riluzole inhibits Na-azide-induced mitochondrial Ca^2+^ signaling and it modestly ameliorate MN degeneration by inhibition of excitotoxicity trigger by [Ca^2+^]_i_ eﬄux.

### Impact of Riluzole on Metabolic State of HMNs in Presence of Sodium Azide-Induced Mitochondrial Respiratory Chain Inhibition and Excitotoxicity

**FIGURE 4:**
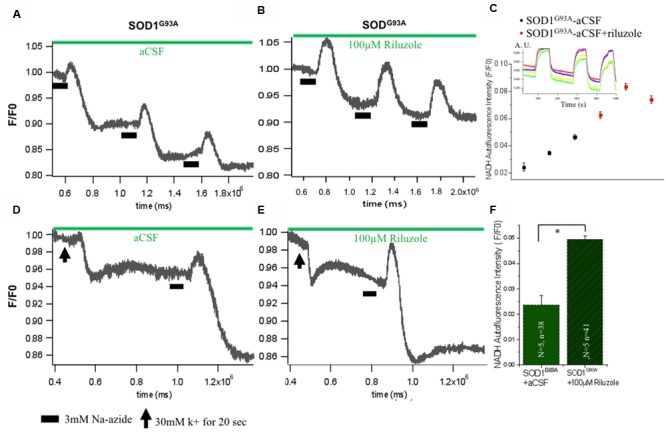
**Impact of riluzole on metabolic state of HMNs measured by NADH fluorescence. (A)** Mitochondrial activity of SOD1^G93A^ mice HMNs was inhibited by 3 mM Na-azide in presence of aCSF. **(B)** Mitochondrial activity of SOD1^G93A^ mice HMNs was inhibited by 3 mM Na-azide in presence of 100 μM riluzole. **(C)** NADH fluorescence intensity profile of HMNs (*n* = 5) measured in a single slice in aCSF (black) and in presence of riluzole (red) after three consecutive application of 3 mM Na-azide. Time series experiment for single measurement shown in inset. **(D)** NADH fluorescence after K^+^ induced plasma membrane depolarization and Na-azide-evoked NADH response in HMNs of SOD1^G93A^ mice in presence of aCSF. **(E)** NADH fluorescence after K^+^ induced plasma membrane depolarization and Na-azide-evoked NADH response in HMNs of SOD1^G93A^ mice in presence of 100 μM riluzole. Horizontal black bars indicate the duration of application of 3 mM Na-azide. The black arrow indicates the 30 mM K^+^ induced plasma membrane for 30 s. **(F)** Graphical representation of the effect of 3 mM Na-azide in presence of aCSF (*N* = 5 slice; *n* = 38 HMNs) and 100 μM riluzole (*N* = 5 slice; *n* = 41) SOD1^G93A^ mice. The data are expressed as mean ± SD; N, number of slices/experiments, n, number of cells. ^∗^*p* < 0.05.

Mitochondrial ETC associated proton eﬄux causes accumulation of negative charges in the matrix and electrochemical gradient across the mitochondrial membrane. Intrinsic NADH fluorescence in MNs has been shown to serve as a valuable tool to characterize the metabolic signature of intrinsic energy profiles. Accordingly, evaluation of intrinsic NADH fluorescence after inhibition of mitochondrial complex IV with Na-azide indicated that the mitochondrial respiratory chain was significantly disturbed in SOD1 G93A animals which is rescues in acute condition by riluzole by yet unknown mechanism (**Figure 4**). To confirm that impact of riluzole on mitochondrial NADH fluorescence, experiments were performed, in which respiratory chain activity of HMNs was inhibited by 3 mM Na-azide in absence (aCSF; **Figure 4A**) and presence of riluzole (100 μM riluzole; **Figure 4B**). Kinetic profile of HMNs (*n* = 5) NADH fluorescence in single slice in aCSF (black) and in presence of riluzole (red) shown in **Figure 4C** for three consecutive application.

To analyze the comparative efficiency of mitochondria of SOD1^G93A^ mice, HMNs were evoked by a 30 mM K^+^ depolarizing stimulus. When SOD1^G93A^ mice HMNs were exposed to 30 mM K^+^ for 20 s, there is immediate and slow plateau shape decrease in the NADH fluorescence and subsequently returned to the basal level. To determine whether the mitochondrial depolarization by 30 mM K^+^ has any apparent influence on the lifetime of the NADH fluorescence and riluzole still rescues HMNs metabolism, 100 μM riluzole was added to the aCSF superfusate after K^+^-induced depolarization (**Figures 4D,E**). Application of 3 mM Na-azide after 20 s depolarization induced by K^+^ resulted in an increase of NADH fluorescence of 0.024 ± 0.003 and 0.049 ± 0.001 in the presence of aCSF (*N* = 5 slice; *n* = 38 HMNs) and aCSF+100 μM riluzole (*N* = 5 slice; *n* = 41 HMNs), respectively, in SOD1^G93A^ mice (**Figure 4F**). Following previous trends there was a slight increase in the NADH fluorescence in presence of 100 μM riluzole but there was no significant difference in NADH fluorescence mean value after 3 mM Na-azide-evoked responses (normalized, *F*/*F*_0_) post K^+^-induced depolarization with or without 100 μM riluzole.

## Discussion

We have shown earlier that [Ca^2+^]_i_ in HMNs increases in response to stimulation with mitochondrial electron transport complex IV inhibitors Na-azide/cyanide and differentially regulated in WT and SOD1^G93A^ mice and cell culture model of SOD1 (Bergmann and Keller, 2004; Jaiswal and Keller, 2009; Jaiswal et al., 2009). Given that riluzole and melatonin has been shown to avert excitotoxicity induced cell death by various mechanism, including oxidative stress and calcium signaling (Wang, 2009; Mazzone and Nistri, 2011; Jaiswal, 2012; Geevasinga et al., 2016; Gutierrez et al., 2016; Reiter et al., 2016), inhibition of persistent sodium currents and Trpm4 (Simard et al., 2012), Trpm4 mediated increase of Ca^2+^ influx (Weber et al., 2010) via Ca^2+^ CRAC and *K*_ca_ channels (Dolmetsch and Lewis, 1994), we examined whether riluzole and melatonin affects mtSOD1-mediated cell death and excitotoxicity and improve cell survival. Furthermore, since Na^+^-azide blocks the mitochondrial ETC in HMNs of ALS, we further analyzed whether riluzole and melatonin protect the HMNs from Na-azide induced MN death induced by excitotoxicity and mediated by inhibition of [Ca^2+^]_mito_ signaling cascades. The effect of drug riluzole and melatonin on the excitotoxicity-induced cell death and [Ca^2+^]_i_ concentration induced by mitochondrial blocker was measured, and the impact of riluzole and melatonin-modulated Ca^2+^ influx and Ca^2+^ release were evaluated. We found that riluzole but not melatonin, moderately inhibits excitotoxicity-induced [Ca^2+^]_mito_ signaling and thereby modestly protects the cell death in WT and SOD1^G93A^ cell culture model of ALS and SOD1^G93A^ mice model (**Figures 1–3**). Previously, it was shown that melatonin enhance mitochondrial ETC I and IV and thereby improving mitochondrial respiration, ATP synthesis and energy metabolism under stress circumstances (Leon et al., 2005). However, in our experiments melatonin fail to show neuroprotective action on either cell death (**Figures 1** and **2**) or Na^+^-azide induced [Ca^2+^]_i_ signaling (data not shown) in adult WT and corresponding symptomatic SOD1^G93A^ mice. Our results indicate that the melatonin mitochondrial neuroprotective action might be governed by respiratory complex I inhibition or by yet unknown unidentified mechanism(s). We therefore believe that the riluzole-targeted inhibition of mitochondrial signaling could be beneficial in ALS. In this paper, we provide evidence using three independent methods about inhibition of depolarization-evoked calcium transients and excitotoxcity in healthy and diseased MNs and cell death/survival assay in cell culture model of MNs by neuropharmacological substrates riluzole to protects cells against excitotoxic damage and hence its therapeutic effects in ALS. These observations may be relevant to understand the vulnerability of MNs to the excitotoxic insult that may contribute to the aetiopathology of ALS.

We found a substantial increase in cell viability and inhibition of excitotoxicity induced cell death in MNs by riluzole but not by melatonin (**Figures 1** and **2**), thus providing evidence for the efficacy of riluzole in *in vitro* transfected cell culture model in the treatment of ALS. Riluzole has been shown to alleviate neurological symptoms in the SOD1^G93A^ Tg mouse model of ALS by suppressing oxidative stress (Seo et al., 2015). Indeed, we observed that riluzole reduced Na-azide induced and SOD1^G93A^-mediated cell death (**Figure 1**) and increase cell viability (**Figure 2**) in SH-SY5Y^WT^ and SH-SY5Y^G93A^ cells, suggesting that mitochondrial excitotoxicity and ROS is associated with the mechanism of SOD1^G93A^-induced cytotoxicity. However, compared to riluzole, melatonin fail to prevent Na-azide induced and SOD1^G93A^-mediated cell death (**Figure 1**) and there is no significant increase in cell viability (**Figure 2**) in SH-SY5Y^WT^ and SH-SY5Y^G93A^ cells, suggesting that mechanism of cell death protection by melatonin might adopt different cellular mechanism as it did not affect cell viability induced by Na-azide induced ROS generation through mitochondrial inhibition.

Furthermore, we demonstrated that the inhibition of peak amplitudes of [Ca^2+^]_i_ in symptomatic SOD1^G93A^ and adult WT mice is significant when measured with application of Na^+^-azide together with riluzole compared to of Na^+^-azide alone (**Figure 3**, *p* < 0.05). The overall inhibition of the rise in peak [Ca^2+^]_i_ transients in the presence of riluzole after a Na^+^-azide application in adult WT and symptomatic SOD1^G93A^ might be due to the potential inhibition of complex IV and decrease in the entry of Ca^2+^ through VDCC. Additionally, in symptomatic SOD1^G93A^ mice, the presence of riluzole slightly diminished the Na^+^-azide induced [Ca^2+^]_mito_ increase and delayed the baseline recovery by up to 30–60 s. Our results therefore indicate that riluzole’s moderate inhibition of [Ca^2+^]_mito_ signaling may be because of a partial blockade of intracellular Ca^2+^ release from the mitochondria. Our finding further supports the assumption that the riluzole ameliorates excitability and Ca^2+^ signaling and this pathway may have role in its manifold effects on mitochondria mediated cellular metabolism in ALS.

NADH fluorescence is a good experimental measure to monitor the metabolic profile of the MNs. In the intact HMNs besetting the mitochondria by Na-azide adversely affects the production of NADH in the mitochondrial metabolism. Our data shows slight increase in the NADH fluorescence in presence of 100 μM riluzole compared to aCSF but there was no significant difference in NADH fluorescence mean value (**Figure 4**). This implies that there is a significant mitochondrial metabolism dysfunction and mitochondria in SOD1^G93A^ mice might not able to sequester enough calcium after K^+^-induced depolarization (dysfunctional Ca^2+^ accumulation activity) or Na-azide-evoked release response is hampered in HMNs of SOD1^G93A^ mice.

**FIGURE 5:**
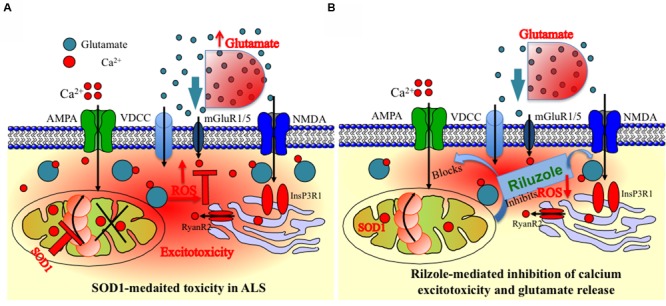
**Schematic presentation of the model of mitochondria-mediated sodium-azide induced excitotoxicity inhibition by Riluzole in ALS. (A)** Mitochondrial disturbance and calcium excitotoxicity associated with SOD1 mediated toxicity are predicted to be the key trigger to ALS etiology. The production of ROS by disturbed mitochondrial metabolism can be readily cytotoxic as ROS can destroy the membrane integrity by averting the specificity of membrane channels or triggering the opening of particular leaky channels as well as by destroying the lipid components of the membrane. Mitochondrial inhibition additionally decreases cellular ATP levels, and this further enhances accumulation of intracellular Ca^2+^. In addition, inhibition of the respiratory chain furthermore decreases the ΔΨm leading to reduced Ca^2+^ uptake into the mitochondrial matrix and potential release of Ca^2+^ into the cytoplasm leading to Ca^2+^ excitotoxicity. Furthermore, it is also hypothesized and estimated that ROS produced in MNs can escape into the extracellular environment and can damage the glutamate transporters on astrocytes. **(B)** An event following a mitochondrial malfunction in MNs inhibits complex IV of the ETC which leads to ROS generation seen in SOD1-mediated toxicity can be chemically trigger by Na-azide. This phenomenon can be partially reversed by blocking ROS production by riluzole in MNs or by inhibition of Ca^2+^ eﬄux at synapse sites. In addition, ROS generated via mitochondria are mostly neutralized by the inhibition of glutamate release trigger by riluzole. The impact of ROS for the cell becomes more severe when mitochondria are placed cardinally to buffer the calcium and to control the subsequent metabolic pathways, since an uncontrolled elevation in the cytosolic Ca^2+^ can lead to immediate cell death. The observed schematic diagram provide a potential mechanism of how mitochondrial inhibition can lead to selective MN degeneration and reverse by riluzole.

Overall, our results indicate that the inhibition and stabilization of Ca^2+^ transient by riluzole under physiological situation, e.g., diffusion-restricted and in homeostatically controlled mitochondrial domains might indeed have numerous functional advantages (**Figure 5**). Accordingly, drugs targeting protection of mitochondrial function and homeostasis could be useful in various forms of ALS by utilizing multidrug therapy where combined pharmacological intervention target different domains of mitochondria and Mito-cycle which ameliorates excitotoxicity both at the MNs and at the neighboring cells and most likely prolong survival of ALS patients. Our experiments promise a limited but effective and methodological way to test the multiple drugs and their targets most likely tested with different drug cocktails to check the efficacy of multi-drug therapy. However, more detailed study allowing identification of mechanism and molecular targets would be necessary.

## Author Contributions

MKJ established the experimental protocol, designed the experiments, carried out all of the experiments, analyzed the data, and wrote the manuscript. Bernhard U. Keller provides laboratory support and financial support for research work.

## Conflict of Interest Statement

The author declares that the research was conducted in the absence of any commercial or financial relationships that could be construed as a potential conflict of interest.

## Abbreviations

aCFS: artificial cerebrospinal fluid
ALS: amyotrophic lateral sclerosis
AM: acetoxy methyl ester
CCD: charge coupled device
CRAC: Ca^2+^ release-activated Ca^2+^
DMSO: dimethyl sulfoxide
ER: endoplasmic reticulum
ETC: electron transport chain
F: fluorescence
fALS: familial amyotrophic lateral sclerosis
HMNs: hypoglossal motoneurons
MNs: motor neurons
NADH: nicotinamide adenine dinucleotide
ROI: regions of interest
ROS: reactive oxygen species
SD: standard deviation
SEM: standard error of mean
SOD1: superoxide dismutase 1
WT: wild-type
[Ca^2+^]_i_: cytosolic calcium
[Ca^2+^]_mito_: mitochondrial calcium
ΔΨm: mitochondrial membrane potential.
